# New Discoveries in Retinal Cell Degeneration and Retinal Diseases

**DOI:** 10.3390/biom13071121

**Published:** 2023-07-14

**Authors:** Puran S. Bora

**Affiliations:** Pat & Willard Walker Eye Research Center, Department of Ophthalmology, Jones Eye Institute, 4301 West Markham, University of Arkansas for Medical Sciences, Little Rock, AR 72205, USA; pbora@uams.edu; Tel.: +1-(501)-352-7191

Age-related macular degeneration (AMD) has two phenotypes: dry AMD and wet AMD. The dry type is characterized by drusen formation and if left untreated can cause wet AMD, which is characterized by neovascularization from existing choroidal vessels. Age-related macular degeneration (AMD) is a complex disease; several other factors (other than complement factors) are responsible for its pathogenesis. *Biomolecules* selects a few special topics to investigate every year. The Editor invites authors working on that particular topic to publish original papers, review articles and short papers. I am the Editor for “New Discoveries in Retinal Cell Degeneration and Retinal Diseases”, a Special Issue of *Biomolecules*.

The review articles in this Special Issue highlight the mechanisms of complement proteins that are involved in the pathogenesis of AMD and current novel therapeutic approaches for the treatment of neovascular age-related macular degeneration [1,2,3,4,5,6,7]. Connor et al. [8] have shown that adiponectin (APN) peptide1 can inhibit new vessel growth in mouse and rat models of choroidal neovascularization (CNV) or wet AMD by 75 percent or more. APN peptide 1 is an anti-inflammatory peptide and inhibits dry AMD. Peptide 1 is derived from the globular region of APN protein and a potent inhibitor of AMD [8]. Currently, we are studying the therapeutic use of this peptide in the treatment of AMD and other eye diseases. One of the original papers published in this Special Issue describes the role of this peptide when inserted into AAV before injecting it into a mouse’s eyes [8]. AMD and retinopathy of prematurity are both associated with altered circulating APN levels and APN variant distributions. In the experiments done by other investigators, APN has been shown to inhibit retinal and CNV defects. As a key glucose and lipid modulator, APN may re-establish metabolic balance. Intervention with ω-3 LCPUFA and derivatives of fibric acid enhance levels of APN in the blood. Exercise may induce positive production of APN systemically as well as locally and plays a protective role in several eye diseases, such as DR, AMD, RP, glaucoma and light-induced retinal degeneration. Further studies are needed to clarify the role of APN/AdipoRs in DR and CNV as well as their underlying molecular mechanisms to better understand both the experimental and clinical impact of this pathway [9].

Other articles and original papers published in this Special Issue highlight the research on retinal pigmented epithelium, optic neuropathy, retinal ganglion cells and other retinal diseases. Five original articles and eight review articles are included altogether. I will briefly describe the highlights of these published articles. Mitchell and Chacko [10] described the role of amiodarone in oculo-neuropathy. Amiodarone is a class III antiarrhythmic agent with characteristics of class I, II and IV antiarrhythmics, and it is widely prescribed for the management of atrial fibrillation and ventricular fibrillation/tachycardia. Alonso et al. [11] highlighted the role of fibrillin in the retina. They found that fibrillin-1/MAGP1 performs essential functions in arteriolar integrity and mutant fibrillin-1-induced defects can be prevented or partially rescued pharmacologically. These new findings could have implications for people with Marfan syndrome. Tsun-Kang Chiang et al. [12] reviewed the use of visual electrophysiology to monitor the retinal and optic nerve toxicity of medications. Lambiri and Levin’s findings [13] correlate well with clinical observations of central loss of the visual field, visual acuity and color vision in LHON, and may serve as an in-silico platform for modeling the mechanism of action for new therapeutics. Haider et al. [14]. explored the role of cGMP signaling in the different cell types of the NVU and investigated the potential links between cGMP signaling, breakdown of neurovascular function and glaucoma pathology. Harmening et al. [15] investigated the use of the SB100X transposase delivered as mRNA and showed that ARPE-19 cells as well as primary human RPE cells were successfully transfected with the Venus or the PEDF gene, followed by stable transgene expression. In human RPE cells, secretion of recombinant PEDF can be detected in cell cultures up to one year later [10]. Non-viral ex vivo transfection using SB100X-mRNA in combination with electroporation increases the biosafety of the gene therapeutic approach to treating nvAMD while ensuring high transfection efficiency and long-term transgene expression in RPE cells. Shan et al. [16], in their review, summarize the incidence, epidemiological characteristics, retinal pathogenesis and accompanying ocular lesions of ARD’s related dry eye, emphasizing the potential role of dry eye in recognizing and monitoring this condition among ARD patients. Pasak et al. [17] showed in their article that at a protein level, CORE2, a subunit of RCC III, and DRP1 were significantly decreased in the neuroretina. Drp1 and Opa1, protein-encoding genes responsible for mt quality control, were decreased in the RPE and neuroretina of most samples. They concluded that the eyes of the minipig can be considered a potential RI model for studying mt dysfunction in this disease. Strategies targeting mt protection are promising for delaying acute damage and onset retinal ischemia [17].

Successful Special Issues are great platforms for the discussion of important and pressing issues, allowing the public to read about diseases and enhance their knowledge in this field. If AMD is not treated, it can cause permanent loss of vision. High-impact journals like *Biomolecules* may offer Special Issues in a book format and may offer the public to have easy access of the Journal/Book to read. Some of the original papers and review articles of this Special Issue have already benefited the people affected by AMD and other retinal diseases.
